# The complete mitochondrial genome of *Achatinella sowerbyana* (Gastropoda: Pulmonata: Stylommatophora: Achatinellidae)

**DOI:** 10.1080/23802359.2016.1219631

**Published:** 2016-09-02

**Authors:** Melissa R. Price, Zac H. Forsman, Ingrid S. Knapp, Robert J. Toonen, Michael G. Hadfield

**Affiliations:** a Kewalo Marine Laboratory, Pacific Biosciences Research Center, University of Hawai‘i at Mānoa, Honolulu, HI, USA;; b Department of Natural Resources and Environmental Management, University of Hawai‘i at Mānoa, Honolulu, HI, USA;; c Hawai‘i Institute of Marine Biology, University of Hawai‘i at Mānoa, Kane‘ohe, HI, USA

**Keywords:** Endangered species, Hawaiian tree snail, mitogenome, Mollusca, RADseq

## Abstract

In this study, we report the complete mitochondrial genome sequence of *Achatinella sowerbyana*, an endangered Hawaiian tree snail. The mitogenome is 15,374 bp in length and has a base composition of A (36.4%), T (42.7%), C (9.1%), and G (11.8%). Similar to other pulmonates, it contains 13 protein-coding genes, 2 ribosomal RNA genes, and 22 transfer RNA genes. The gene order is the same as its sister species, *A. mustelina*. To our knowledge, this is the second mitochondrial genome sequenced within the superfamily Achatinelloidea, and will aid in the examination of the genus *Achatinella*, part of a spectacular radiation in the Hawaiian Islands.

*Achatinella sowerbyana*, endemic to the Ko‘olau Mountain Range on the island of O‘ahu, is part of a spectacular radiation with more than 41 species in the genus (Pilsbry & Cooke 1912–1914). Habitat loss, predation by introduced species, and over-harvesting by collectors led to the extinction of at least 30 species of *Achatinella*, and resulted in the declaration of all remaining species in the genus as Endangered (Hadfield & Mountain 1980; Hadfield 1986; U.S. Fish and Wildlife Service 1981). Of those that remain, *A. sowerbyana* is the second most abundant, with less than 10 populations in the wild.

We sequenced the complete mitochondrial genome of *A. sowerbyana* (GenBank accession number KX356680). Small tissue samples were collected from 26 individuals at a single study site (21.53742, -157.92099), using non-lethal methods, and preserved in 100% ethanol until DNA extraction (Thacker & Hadfield 2000). DNA was individually extracted from tissue samples using a DNeasy Blood and Tissue Kit (Qiagen, Valencia, CA) according to the manufacturer’s protocol. Extracted DNA was quantified using the Biotium (Hayward, CA) AccuClear Ultra High Sensitivity dsDNA quantitation kit using seven standards. Equal quantities of DNA from each individual were pooled to a total of 1 μg, and a library was prepared for genome scanning using the ezRAD protocol (Toonen et al. 2013) version 2.0 (Knapp et al. in press). The sample was digested with the frequent cutter restriction enzyme DpnII from New England Biolabs^®^ (Ipswich, MA) and fragments between 300 and 700 bp in length prepared for sequencing on the Illumina^®^ MiSeq using the Kapa Biosystems (Wilmington, MA) Hyper Prep kit. The sample was amplified to generate 1 μg of adapter-ligated DNA, then validated and quantified to ensure equal pooling on the MiSeq flow cell, using a Bioanalyzer and qPCR. Quality control checks and sequencing were performed by the Hawaii Institute of Marine Biology Genetics Core Facility.

We obtained 5,737,666 sequences. Reads were paired, then mapped to the mitogenome of *A. mustelina* (Price et al. 2016) using Geneious 6.0 (Biomatters, Newark, NJ). In total, 5442 reads, or about 0.1% of reads, mapped to the mitochondrial genome, with coverage ranging from 1× to 844× per site (99 ± 172). Coverage was lower in regions that had multiple cut sites within a 200 bp span, and in regions that had greater than 2000 bp between cut sites. Annotation of mitochondrial elements was carried out with DOGMA (Wyman et al. 2004) and MITOS (Bernt et al. 2013).

The mitogenome of *A. sowerbyana* is similar to those of other pulmonates (Figure 1, White et al. 2011), with 13 protein-coding genes, 2 rRNA genes, and 22 tRNA genes, and a base composition of A (36.4%), T (42.7%), C (9.1%), and G (11.8%). The mitogenome is 15,374 bp in length, nearly 1000 bp smaller than that of *A. mustelina* (Price et al. 2016). Most of the difference in length is accounted for in the intergenic regions. This is the second mitogenome sequenced within the superfamily Achatinelloidea, and will aid in the evolutionary studies of the spectacular radiation of Hawaiian tree snails across the Hawaiian Islands.

**Figure 1.**
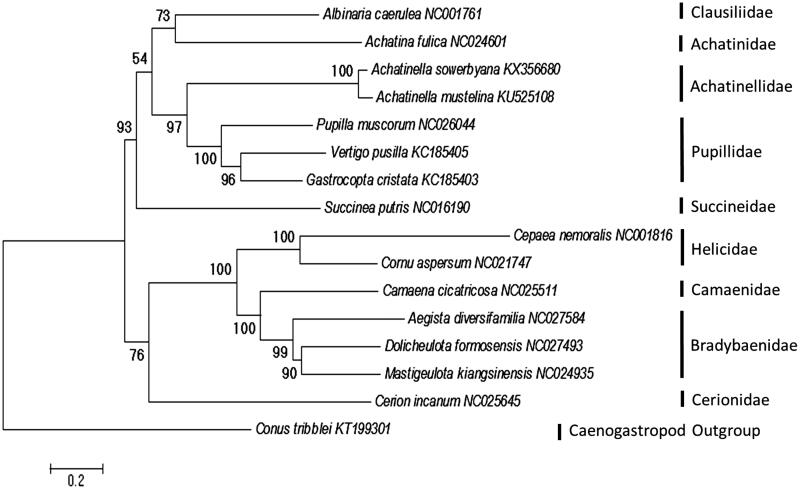
Placement of *Achatinella sowerbyana* among Stylommatophoran snails. Alignments, model tests, and maximum-likelihood analyses were performed using MEGA version 6.2 (Tamura et al. 2013). The 13 protein-coding mitochondrial gene sequences were individually translated into amino acid sequences, then aligned using ClustalW in MEGA version 6.2. Default settings were used with the following exception: the multiple alignment parameters were changed to a gap opening penalty of 3.0; and the gap extension penalty was set to 1.8. The amino acid substitution model was found to be LG + G + I + F using the Akaike Information Criterion (AIC). Maximum-likelihood analysis of the amino acid sequences was run using the identified model, with bootstrap support values based on 1000 replicates. The resulting tree shows similar relationships to previous studies (Hatzoglou et al. 1995; Grande et al. 2004; Knudsen et al. 2006; White et al. 2011; Yamazaki et al. 1997; Gaitán-Espitia et al. 2013; He et al. 2014; Wang et al. 2014; Huang et al. 2015; Deng et al. 2016; Price et al. 2016).
